# Pulsed Electromagnetic Field Therapy in People with Knee Osteoarthritis: A Systematic Review and Meta-Analysis

**DOI:** 10.3390/medicina62040677

**Published:** 2026-04-02

**Authors:** Yu-Shan Chang, Chieh-Yu Lin, Wan-Chi Huang

**Affiliations:** Department of Physical Medicine and Rehabilitation, Changhua Christian Hospital, Changhua 50006, Taiwan; sandra860829@gmail.com (Y.-S.C.); channy8411@gmail.com (C.-Y.L.)

**Keywords:** knee osteoarthritis, osteoarthritis, pulsed electromagnetic field, pulsed electromagnetic field therapy, systematic review

## Abstract

*Background and Objectives*: Knee osteoarthritis (KOA) is a major cause of global disability. The efficacy of a non-invasive treatment, pulsed electromagnetic field (PEMF) therapy, remains debated. This systematic review and meta-analysis evaluate PEMF’s effectiveness on KOA, exploring the influence of device parameters. *Materials and Methods*: We systematically searched PubMed, Embase, and the Cochrane Library for randomized controlled trials (RCTs) from 2015 to 2025. Nine RCTs with a total of 457 patients were included. Primary outcomes were pain (Visual Analog Scale—VAS) and function (Western Ontario and McMaster Universities Osteoarthritis Index—WOMAC). Data were pooled using a random-effects model with subgroup analyses based on PEMF amplitude and frequency. *Results*: No significant improvement in VAS pain or total WOMAC scores was found at one month. However, time-dependent effects were observed. WOMAC-pain improved significantly at 18–21 days (MD = −1.63, 95% CI: −2.43 to −0.82, I^2^ = 28%) but not at one month. Conversely, WOMAC-stiffness (MD = −1.11, 95% CI: −1.386 to −0.85, I^2^ = 0%) and daily activity (MD = −3.39, 95% CI: −4.81 to −1.97, I^2^ = 0%) improved significantly only at the one-month. Objective functional measures did not improve, and the overall risk of bias across studies was high. The efficacy of PEMF is also influenced by the amplitude and frequency. *Conclusions*: PEMF efficacy for KOA is nuanced, with benefits dependent on timing and device parameters. High frequency gives fast pain relief; high amplitude builds function. Though statistically significant, these improvements may not reach thresholds for clinical meaningfulness. Significant heterogeneity in treatment protocols is a major barrier to clear conclusions. Standardized, large-scale RCTs are needed to determine optimal parameters and confirm PEMF’s clinical role.

## 1. Introduction

Osteoarthritis (OA) represents the most prevalent form of arthritis and a leading cause of pain and disability across the globe [1]. The condition has reached epidemic levels, with the Global Burden of Disease (GBD) 2021 study estimating approximately 606.9 million cases globally [1]. While OA can manifest in any joint, disease in the hip and knee is responsible for the most significant societal costs [2]. The biomechanical hallmark of knee osteoarthritis (KOA) involves altered joint loading and articular cartilage degradation, which precipitates subchondral bone sclerosis and synovial inflammation [3,4]. This structural deterioration mechanically triggers nociceptive pathways, leading to the characteristic persistent pain and reflex muscle inhibition that progressively restrict physical function [3,4]. The condition is clinically characterized by persistent pain, joint stiffness, and loss of physical function, which impair patients’ ability to perform daily activities. Indeed, 80% of OA patients report limitations in movement, and 25% require assistance for major life activities [5]. The functional decline associated with KOA frequently leads to reduced physical activity, leading to social isolation, psychological distress, including anxiety and depression, and poor overall well-being [6,7,8]. Before considering surgical options, established conservative treatment for osteoarthritis forms the cornerstone of management. These interventions encompass a multidisciplinary approach, including structured exercise programs, knee bracing, physical modalities, and pharmacological therapies aiming to alleviate symptoms and preserve joint function [9,10,11,12]. Accordingly, evidence-based guidelines from leading organizations—including Osteoarthritis Research Society International (OARSI), American College of Rheumatology/Arthritis Foundation (ACR/AF), and European Alliance of Associations for Rheumatology (EULAR)—uniformly advocate for a foundational treatment strategy centered on core non-pharmacological interventions [13,14,15].

Pulsed electromagnetic field (PEMF) therapy is a non-invasive and non-thermal modality that employs low-frequency magnetic fields to promote tissue healing [16]. The technology operates via a control unit that sends specific electrical currents through insulated coils, thereby generating a pulsed magnetic field [17]. At the biological level, these electromagnetic pulses are thought to trigger a physiological cascade [18]. The proposed effects include optimizing cellular metabolism, enhancing local microcirculation and tissue oxygenation, and stimulating the body’s natural regenerative pathways [16].

Although supported by a strong preclinical rationale, the clinical efficacy of PEMF therapy for KOA remains a subject of considerable debate and controversy [19,20,21,22,23]. The literature is further limited by a lack of subgroup analyses investigating how outcomes may be influenced by device parameters such as frequency and amplitude. Accordingly, this systematic review aims to synthesize the available evidence using a structured PICO framework: In adult patients with knee osteoarthritis of any severity (Population), what is the efficacy of PEMF therapy, either alone or as an adjunct to standard care (Intervention), compared to sham PEMF, active physical therapy modalities, or standard exercise (Comparator), for managing pain, physical function, and quality of life at short-term follow-ups (Outcomes)? We will also conduct a subgroup analysis to explore the differential effects of PEMF amplitude and frequency.

## 2. Materials and Methods

### 2.1. Search Strategy

We conducted this systematic review according to a prespecified protocol registered with PROSPERO (CRD420251143716), and this report adheres to the PRISMA 2020 statement (PRISMA checklist is presented in Table S1) [24]. To identify eligible RCTs, a comprehensive literature search was executed, including PubMed, Embase, and the Cochrane Library. The search strategy was limited to articles published from 1 January 2015 to 1 September 2025, that evaluated the efficacy of PEMF therapy in patients with KOA. The search terms used included: (“osteoarthritis, knee” OR “knee osteoarthritis” OR “gonarthrosis” OR “degenerative joint disease”) AND (“electromagnetic fields” OR “pulsed electromagnetic field” OR “PEMF”). The search was limited to English-language publications. In addition, we performed a manual search of the reference lists from all included articles and relevant systematic reviews to ensure literature saturation. The study selection process was conducted in duplicate by two independent authors. The detailed search strategy is presented in Table S2.

### 2.2. Inclusion Criteria

Eligible studies were required to meet the following criteria: (1) they were full-text RCTs; (2) adult patients (aged 18 years or older); (3) participants diagnosed with KOA of any severity who received PEMF therapy; and (4) the control group received sham PEMF, another active physical therapy modality, or exercise. If co-interventions (e.g., standard physical therapy or exercise) were used, they had to be applied equally to both the experimental and control groups.

### 2.3. Exclusion Criteria

Studies were also excluded if they were: (1) non-RCTs, (2) non-English publications; and (3) involving participants younger than 18 years.

### 2.4. Primary Outcome

We extracted data on three primary outcomes: pain, function, and lower limb strength. Pain was assessed subjectively using the Visual Analog Scale (VAS), with scores ranging from zero to ten and zero to one hundred, respectively. Function was evaluated using the Western Ontario and McMaster Universities Osteoarthritis Index (WOMAC), from which we extracted the total score and subscores for pain, stiffness, and daily activity. Finally, lower limb strength was assessed using performance-based tests, specifically the Timed Up and Go (TUG) test and the Five Times Sit-to-Stand Test (FTSST).

### 2.5. Data Extraction

Using a standardized form, two authors independently extracted data from each eligible study. The extracted information included (1) study characteristics: design, sample size, and follow-up intervals; (2) intervention details: parameters of the PEMF application and the nature of the control group; and (3) outcome data: summary statistics for pain, function, and strength. For trials with multiple arms, we established a rule to combine similar arms prior to analysis.

### 2.6. Bias Assessment and Quality Classification

The methodological quality of each included RCT was assessed in duplicate by two independent reviewers using the Cochrane risk-of-bias 2.0 (RoB 2) tool, across six domains: (1) randomization process, (2) deviations from intended interventions, (3) missing outcome data, (4) outcome measurement, (5) selection of reported results, and (6) overall bias [25]. Each domain, along with an overall study judgment, was categorized as having a “Low risk,” “Some concerns,” or “High risk” of bias.

### 2.7. Statistical Analysis

We performed all meta-analyses using Review Manager 5.3 (RevMan 5.3). The primary effect measure for continuous outcomes was the mean difference (MD) with its corresponding 95% confidence interval (CI). To preserve clinical interpretability and avoid the potential distortion of data standardization, we chose not to convert different pain scales into a Standardized Mean Difference (SMD). Instead, studies reporting VAS on a 0–10 scale and those using a 0–100 scale were analyzed separately as distinct subgroups. We quantified heterogeneity using the I^2^ statistic. For studies that reported medians or ranges, data were converted to mean and standard deviation (SD) following validated statistical methods [26]. The choice of a random-effects model for pooling was determined a priori to account for expected clinical and methodological heterogeneity. To explore potential sources of heterogeneity, we conducted subgroup analyses based on key PEMF parameters (frequency and amplitude). Additionally, as specific follow-up time points were not defined a priori in the protocol, we categorized outcome data post hoc based on the natural clustering of follow-up periods reported in the included trials (i.e., 18–21 days and one month) to prevent blending heterogeneous temporal effects. Statistical significance was defined as a two-tailed *p*-value less than 0.05.

## 3. Results

### 3.1. Study Identification and Selection

**Figure 1:**
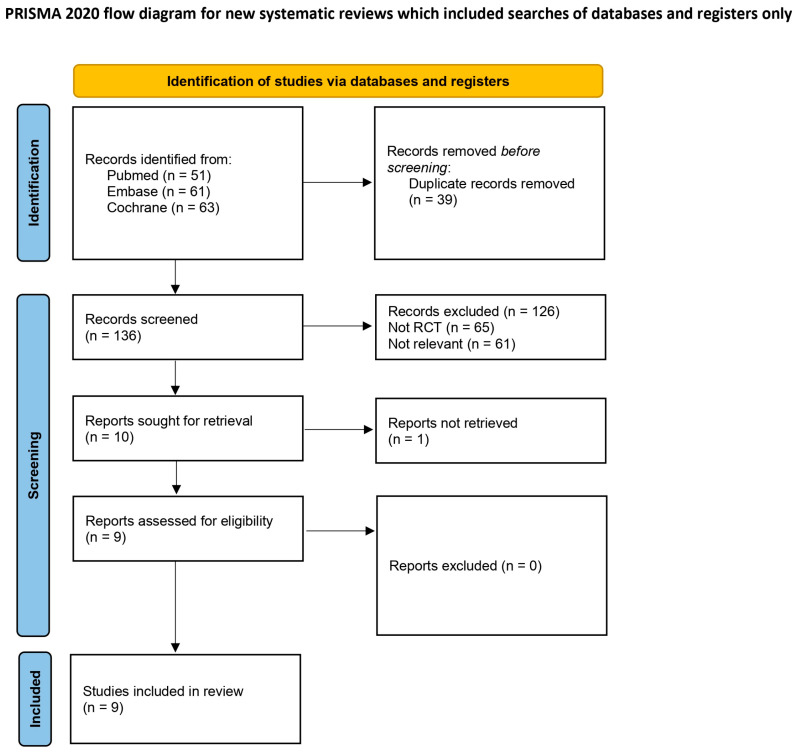
Flow diagram describing the screening and review processes of the meta-analysis [24].

A comprehensive literature search identified 175 potentially relevant records. Following excluding 39 duplicates, 136 records remained for screening. This process resulted in the exclusion of 126 records, primarily due to irrelevant study designs or populations. The remaining 10 articles were assessed for eligibility via full-text review. One of these was excluded as the full-text reports could not be obtained (Table S3) [27]. Ultimately, nine RCTs were included in the final qualitative and quantitative synthesis (Figure 1).

The nine included RCTs contributed a total of 457 patients, with 243 randomized to receive PEMF and 214 to control groups [22,23,28,29,30,31,32,33,34]. Detailed characteristics of the included studies are presented in Table 1. The trials were published between 1 January 2015 and 1 September 2025, and sample sizes varied from 34 to 66 participants. There was considerable heterogeneity in the PEMF application parameters. One study utilized a high-amplitude protocol (>100 mT) and one used a high-frequency protocol (>100 Hz), while the remaining studies employed lower-amplitude and lower-frequency settings. There was variability in the outcomes reported across the nine included trials. Seven studies reporting VAS scores (four on a zero-to-ten scale and three on a zero-to-one-hundred scale). The WOMAC score for function was reported in six studies. Functional performance tests, specifically the TUG and FTSST, were utilized in only three and two of the included trials, respectively.

### 3.2. Methodological Quality of the Included Studies

**Figure 2:**
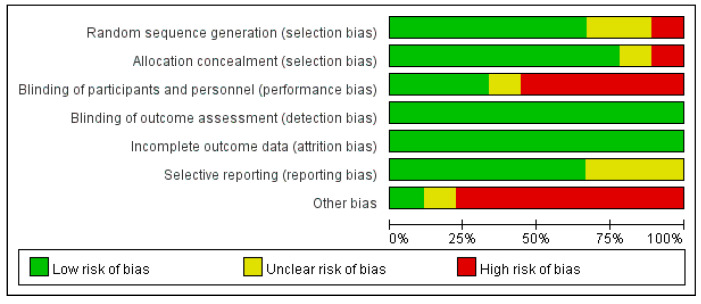
Summary of quality assessment of studies included in the meta-analysis using Cochrane risk-of-bias 2 tool.

**Figure 3:**
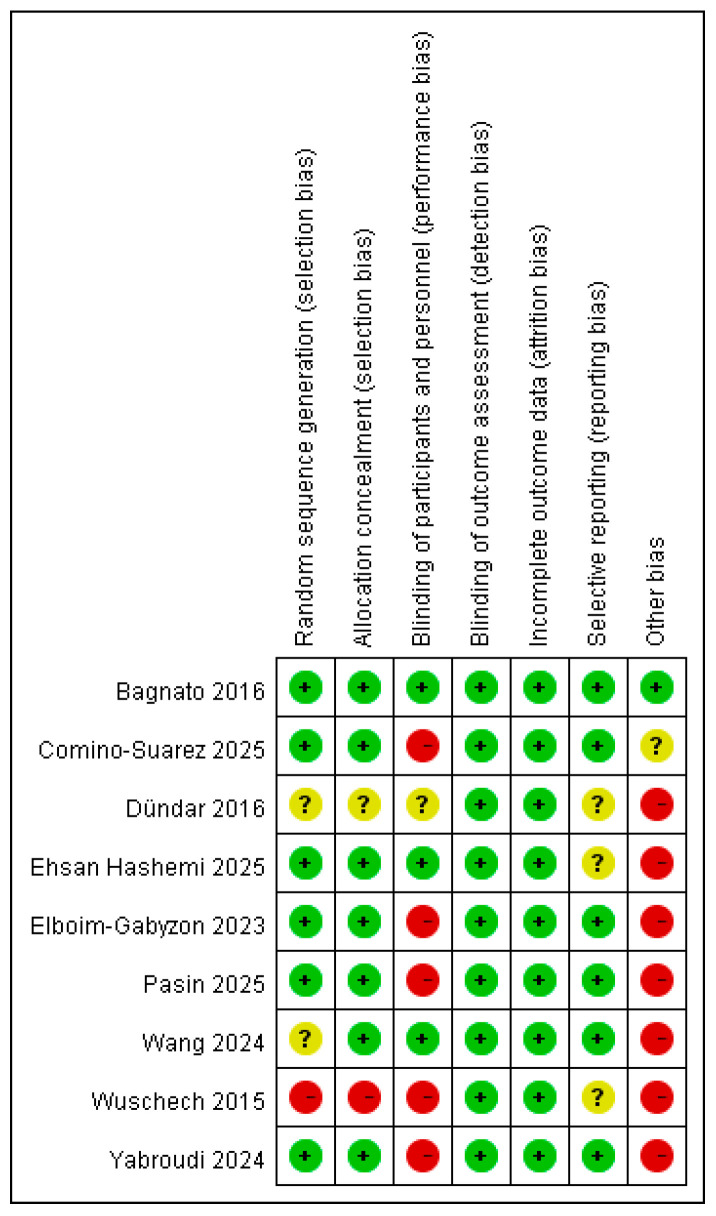
Summary of quality assessment of studies included in the meta-analysis using Cochrane risk-of-bias 2 tool [22,23,28,29,30,31,32,33,34]. Green circles: low risk of bias; yellow circles: unclear risk of bias; red circles: high risk of bias.

The risk of bias of this study is demonstrated in Figure 2 and Figure 3.

#### 3.2.1. Random Sequence Generation

Six of the nine included trials were judged to be at low risk, having adequately described their method of random sequence generation. In contrast, the study by Wuschech et al. (2015) was rated at high risk after its randomization procedure was abandoned partway through the trial, and a majority of the treatment group was assigned non-randomly [28]. The remaining two studies (Dündar et al., 2016; Wang et al., 2024) had a lack of reporting on how the randomization sequence was generated, resulting in an unclear risk of bias [23,29].

#### 3.2.2. Allocation Concealment

Seven of the nine trials were assessed at low risk, having employed adequate concealment methods like sealed opaque envelopes or centralized randomization systems. In contrast, the Wuschech et al. (2015) study was rated at high risk because allocation concealment was breached for a subgroup of participants [28]. A judgment of unclear risk was applied to the Dündar et al. (2016) trial due to the concealment mechanism not being mentioned [29].

#### 3.2.3. Blinding of Participants and Personnel

Five studies were rated at high risk, typically because blinding was impossible when comparing PEMF to other distinct interventions, or because the blinding procedure was documented to have failed [28,30,31,32,33]. One study (Dündar et al., 2016) was assessed as unclear risk due to insufficient reporting on the blinding of the therapist [29].

#### 3.2.4. Blinding of Outcome Assessment

All included studies were rated at a low risk of bias. Every trial confirmed that outcome assessors were blinded to participants’ group assignments, and some studies further strengthened this methodology by employing independent assessors who were not involved in providing treatment.

#### 3.2.5. Incomplete Outcome Data

All trials demonstrated low risk of bias. This was attributed to low participant attrition rates, with several trials reporting no dropouts.

#### 3.2.6. Selective Reporting

Three trials were judged to have unclear risk because they did not mention a pre-registered protocol, and it was not possible to determine whether the reported outcomes were selected based on the results [28,34].

#### 3.2.7. Other Bias

Seven trials were judged to have a high overall risk of bias, due to a combination of issues, such as significant baseline imbalances, uncontrolled co-interventions, or the potential for differential placebo effects. One study was assessed as having unclear risk, primarily because the intervention was administered by three different physical therapists, which could introduce variability despite standardized training.

### 3.3. Outcome

#### 3.3.1. Pain Scores at One Month

**Figure 4:**
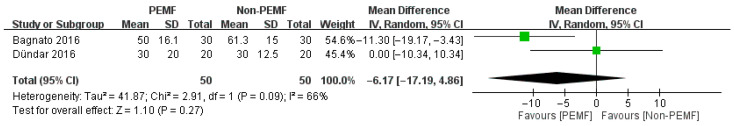
Forest plot of the effects of PEMF at one month on pain score (VAS, 0–100) [22,29].

**Figure 5:**
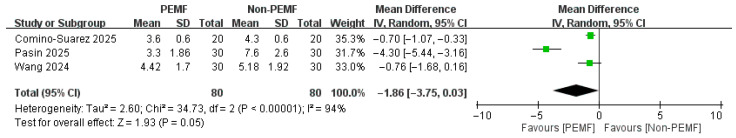
Forest plot of the effects of PEMF at one month on pain score (VAS, 0–10) [23,32,33].

The meta-analysis of pain at the one-month follow-up included five studies with a total of 260 participants. Two studies used a zero-to-one hundred VAS, and others used a zero-to-ten VAS. While patients who received PEMF reported lower pain scores in both subgroups, the differences compared to the control groups were not statistically significant [zero to one hundred: MD = −6.17, 95% CI: −17.19 to 4.86; *p* = 0.27; zero to ten: MD = −1.86, 95% CI: −3.75 to 0.03; *p* = 0.05] (Figure 4 and Figure 5).

#### 3.3.2. WOMAC-Total Score at 18–21 Days and One Month

**Figure 6:**
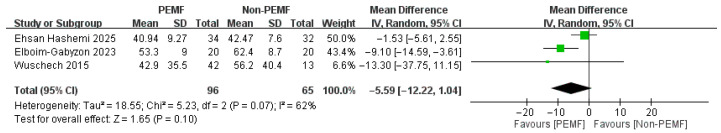
Forest plot of the effects of PEMF at 18–21 days on WOMAC-total score [28,30,34].

**Figure 7:**
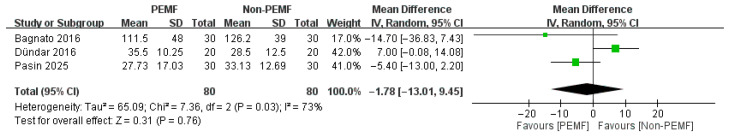
Forest plot of the effects of PEMF at one month on WOMAC-total score [22,29,32].

An analysis of the WOMAC total score was conducted at two separate time points, incorporating data from three studies (*n* = 161) at 18–21 days and three studies (*n* = 160) at one month. At both follow-up periods, patients receiving PEMF therapy showed a trend towards better physical function with lower WOMAC scores, but this improvement was not statistically significant [At 18–21 days: MD = −5.09, 95% CI: −12.22 to 1.04; *p* = 0.10; At one month: MD = −1.78, 95% CI: −13.01 to 9.45; *p* = 0.76] (Figure 6 and Figure 7).

#### 3.3.3. WOMAC-Pain Subscore at 18–21 Days and One Month

**Figure 8:**
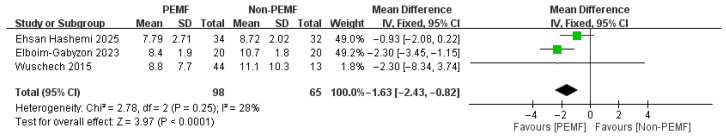
Forest plot of the effects of PEMF at 18–21 days on WOMAC-pain subscore [28,30,34].

**Figure 9:**
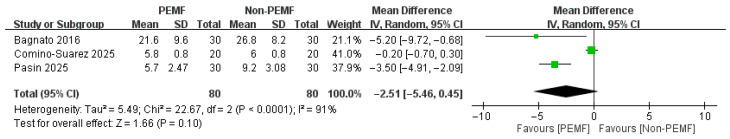
Forest plot of the effects of PEMF at one month on WOMAC-pain subscore [22,32,33].

For the WOMAC-pain subscore, the analysis at 18–21 days revealed a statistically significant reduction in pain for the PEMF group compared to the control group. However, at the one-month follow-up, there was no significant difference in pain scores between the groups [At 18–21 days: MD = −1.63, 95% CI: −2.43 to −0.82; *p* < 0.0001; at one month: MD = −2.51, 95% CI: −5.46 to 0.45; *p* = 0.10] (Figure 8 and Figure 9).

#### 3.3.4. WOMAC-Stiffness Subscore at 18–21 Days and One Month

**Figure 10:**
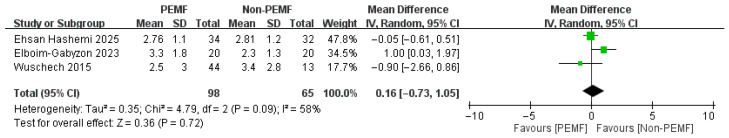
Forest plot of the effects of PEMF at 18–21 days on WOMAC-stiffness subscore [28,30,34].

**Figure 11:**
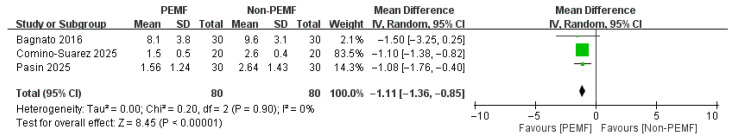
Forest plot of the effects of PEMF at one month on WOMAC-stiffness subscore [22,32,33].

For the WOMAC-stiffness subscore, no significant difference was found between the PEMF and control groups at 18–21 days (MD = 0.16, 95% CI: −0.73 to 1.05; *p* = 0.72). However, at the one-month follow-up, the analysis showed a highly significant reduction in stiffness favoring the PEMF group [At 18–21 days: MD = 0.16, 95% CI: −0.73 to 1.05; *p* = 0.72; at one month: MD = −1.11, 95% CI: −1.36 to −0.85; *p* < 0.00001] (Figure 10 and Figure 11).

#### 3.3.5. WOMAC-Daily Activity Subscore at One Month and 18–21 Days

**Figure 12:**
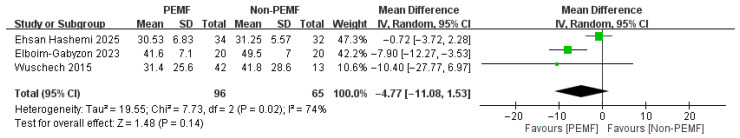
Forest plot of the effects of PEMF at 18–21 days on WOMAC-daily activity subscore [28,30,34].

**Figure 13:**
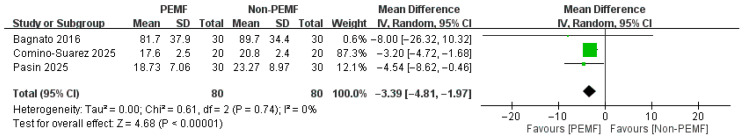
Forest plot of the effects of PEMF at one month on WOMAC-daily activity subscore [22,32,33].

For the WOMAC-daily activity subscore, no significant difference was found between groups at 18–21 days. However, at the one-month follow-up, the analysis revealed a highly significant improvement in daily activity favoring the PEMF group [At 18–21 days: MD = −4.78, 95% CI: −11.08 to 1.53; *p* = 0.14; at one month: MD = −3.39, 95% CI: −4.81 to −1.97; *p* < 0.00001] (Figure 12 and Figure 13).

#### 3.3.6. TUG (Seconds) at One Month

**Figure 14:**
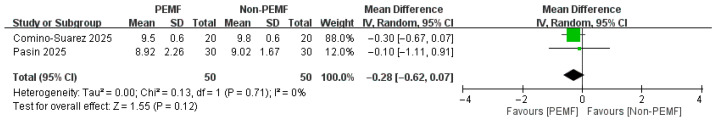
Forest plot of the effects of PEMF at one month on TUG (seconds) [32,33].

For the TUG test at the one-month follow-up, there was no significant difference between the PEMF and control groups [MD = −0.28, 95% CI: −0.62 to 0.07; *p* = 0.12] (Figure 14).

#### 3.3.7. FTSST (Seconds) at Two Months

**Figure 15:**
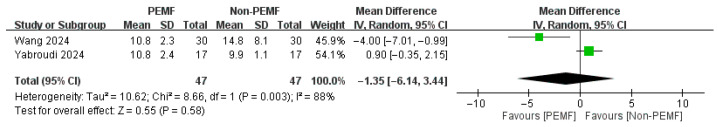
Forest plot of the effects of PEMF at two months on FTSST (seconds) [23,31].

At the two-month follow-up, the analysis of the FTSST showed no significant difference between the PEMF and control groups [MD = −1.35, 95% CI: −6.14 to 3.44; *p* = 0.58] (Figure 15).

### 3.4. Subgroup Analysis

**Figure 16:**
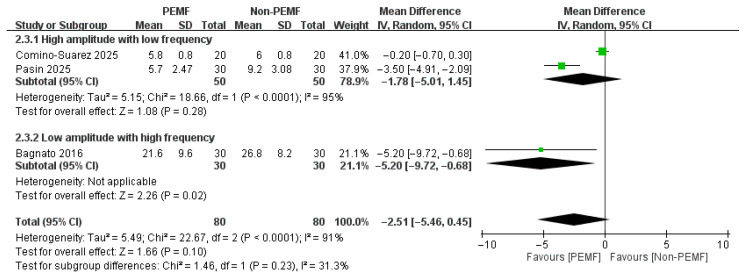
Forest plot of the subgroup analysis. Compare participants using different levels of amplitude (>1 mT vs. ≤1 mT) with different levels of frequency (>100 Hz vs. ≤100 Hz) [22,32,33]. The effects of PEMF at one month on WOMAC-pain subscore.

**Figure 17:**
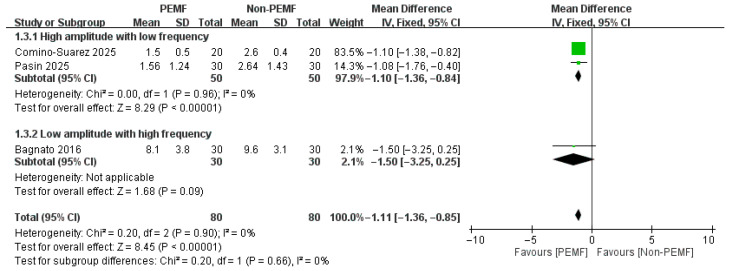
Forest plot of the subgroup analysis. Compare participants using different levels of amplitude (>1 mT vs. ≤1 mT) with different levels of frequency (>100 Hz vs. ≤100 Hz) [22,32,33]. The effects of PEMF at one month on WOMAC-stiffness subscore.

**Figure 18:**
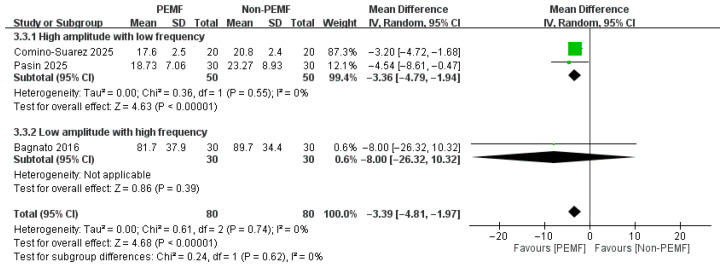
Forest plot of the subgroup analysis. Compare participants using different levels of amplitude (>1 mT vs. ≤1 mT) withlevelserent level of frequency (>100 Hz vs. ≤100 Hz) [22,32,33]. The effects of PEMF at one month on WOMAC-daily activity subscore.

To investigate the influence of device parameters, we conducted a subgroup analysis at the one-month follow-up based on PEMF amplitude (>1 mT vs. ≤1 mT) and frequency (>100 Hz vs. ≤100 Hz). The results showed distinct and opposing effects on WOMAC subscores. Relief in WOMAC-pain was statistically significant only in the low-amplitude, high-frequency subgroup. Conversely, improvements in WOMAC-stiffness and WOMAC-daily activity were significant only in the high-amplitude, low-frequency subgroup (Figure 16, Figure 17 and Figure 18).

## 4. Discussion

### 4.1. Principal Findings

This systematic review examined outcome reporting in RCTs related to PEMF in KOA. While we found no significant improvement in overall pain or total WOMAC scores, a granular analysis showed distinct patterns. A significant reduction in pain was observed early but was not maintained at one month. Conversely, significant improvements in stiffness and daily activity emerged only at the one-month follow-up. Despite these self-reported gains, objective functional measures did not significantly change. Furthermore, subgroup analyses indicate that treatment efficacy is highly dependent on device parameters like amplitude and frequency.

### 4.2. Context with the Existing Literature

The existing evidence on PEMF therapy is extensive, and our findings, with their emphasis on nuance, help to clarify the landscape. At first glance, our lack of an overall significant effect seems to contrast with several major meta-analyses that concluded PEMF is broadly effective for pain and function in KOA [35,36]. However, our positive findings within specific subgroups and time points are actually consistent with this consensus. Our review suggests that the therapeutic benefit is not a simple “yes or no” but is conditional upon factors like treatment duration and device settings. A simplistic pooled analysis can mask these important effects, which may explain why our overall result was not significant while the underlying signals of efficacy were still present.

This interpretation is strengthened when considering studies with divergent results, such as the meta-analysis by Chen et al. [19], which found a benefit for physical function but not for pain or stiffness. This discrepancy is not necessarily a contradiction but rather highlights the critical role of treatment parameters. The substantial heterogeneity in PEMF protocols across the included studies—encompassing wide variations in amplitude, frequency, and treatment duration—significantly complicates the interpretation of our pooled results. This heterogeneity means that a ‘one-size-fits-all’ conclusion about PEMF is difficult to draw, as different protocol combinations likely exert varying degrees of biological influence. Our analysis included newer RCTs and used a different statistical approach (standard mean difference, which is better for comparing different scales), but the most likely reason for the difference is their inclusion of only “classical PEMF therapy.” This suggests that their study pool may have excluded protocols with specific biophysical parameters that are necessary to produce an analgesic effect. Therefore, the divergence in findings across reviews may actually be further evidence for our conclusion: the therapeutic effect of PEMF is highly specific, and its success in a meta-analysis depends critically on the characteristics of the included trials.

### 4.3. Clinical and Mechanistic Implications

While our analysis identified statistically significant effects, it is crucial to assess if they are clinically meaningful. For the WOMAC (zero-to-one-hundred scale), this requires an improvement of approximately 7.8 points for the total score, 8.7 for pain, 20.2 for stiffness, and 14.5 for function [37]. The corresponding minimal clinically important difference (MCID) for the VAS is 19.9 mm [36]. Our pooled MDs, even when statistically significant, did not meet these MCID thresholds. Therefore, it is crucial to clearly distinguish between statistical significance and clinical relevance in this context. This suggests that while PEMF can produce measurable statistical benefits, the average effect size in the existing literature may not yet translate to a change that is clearly perceptible or clinically meaningful to the average patient.

These clinical benefits are supported by a compelling body of preclinical evidence elucidating PEMF’s mechanisms. The therapeutic effects appear to stem from a synergistic triad of actions:

(1) Anti-Inflammatory Modulation: PEMF has been shown to downregulate key pro-inflammatory cytokines (e.g., TNF-α, IL-6) by inhibiting the NF-κB signaling pathway, directly addressing the inflammatory component of OA pain [38].
(2) Chondroprotection: PEMF may exert a disease-modifying effect by stimulating chondrocyte proliferation and the synthesis of essential cartilage matrix components like collagen and aggrecan [39].
(3) Muscle Function Enhancement: Emerging evidence indicates that PEMF, when combined with exercise, can significantly improve knee-extensor and flexor strength. This enhancement of the knee’s musculoskeletal support system is a critical, and perhaps underappreciated, contributor to the functional gains observed in our review [23].

### 4.4. Role of Amplitude and Frequency

Our analysis reveals a distinct dichotomy: low-amplitude (≤1 mT), high-frequency (>100 Hz) signals significantly reduce pain, while high-amplitude (>1 mT), low-frequency (≤100 Hz) signals improve stiffness and function. This hypothesis-generating observation suggests the possibility that the amplitude and frequency of the PEMF signal might influence its primary biological effect and clinical outcome. The potential influence of low-amplitude, high-frequency PEMF on pain might be related to its role as an “informational signal”.

The influence of low-amplitude, high-frequency PEMF on pain is best explained by its role as an “informational signal” that modulates inflammation. This combination is effective at upregulating cell surface adenosine receptors (A2A and A3), reducing the production of cytokines that drive KOA pain [40]. The low amplitude is sufficient for this signaling cascade, while the high frequency provides a rapid, repetitive stimulus, consistent with clinical reports of fast pain relief [41].

Conversely, the effect of high-amplitude, low-frequency PEMF on function could hypothetically act as an “energetic stimulus” that promotes tissue regeneration. High-amplitude fields are theorized to provide the necessary energy to stimulate chondrocyte proliferation and the synthesis of extracellular matrix components like collagen and aggrecan, directly addressing cartilage degradation [42]. This drives the slower, anabolic processes required for structural and functional gains, which also includes improving muscle strength around the joint. However, it is critical to emphasize that these subgroups contained a very limited number of studies (e.g., only one study in the low-amplitude/high-frequency group). Consequently, these mechanistic explanations remain highly speculative and must be interpreted with extreme caution until confirmed by adequately powered, targeted experimental and clinical trials.

### 4.5. Strengths and Limitations

The robustness of our findings is supported by several methodological strengths. By adhering strictly to PRISMA guidelines, employing a comprehensive search strategy across multiple databases, and including only RCTs, we have provided a transparent and high-level synthesis of the current evidence. Furthermore, the formal risk-of-bias assessment allows for a critical appraisal of the quality of the included studies.

Nonetheless, this review has several limitations that temper its conclusions. First, the limited number of high-quality RCTs eligible for inclusion (only nine trials involving a total of 457 patients) fundamentally constrains the statistical power of the analysis. Given this relatively small sample size, our meta-analysis should be viewed somewhat as a pilot synthesis, and the pooled effect estimates must be interpreted with caution. Second, by restricting our search to English-language studies and limiting our database selection to PubMed, Embase, and the Cochrane Library (excluding gray literature, clinical trial registries, and physiotherapy-specific databases such as PEDro or CINAHL), we may have introduced publication bias and potentially missed relevant trials. Third, the focus on short-term outcomes (primarily at 18–21 days and one month) prevents any conclusions about the long-term efficacy or the durability of the treatment effects of PEMF over extended periods. Finally, the necessary conversion of medians and ranges to means and standard deviations may have introduced some statistical imprecision. Future research should prioritize large-scale, long-term RCTs with standardized device parameters and should also systematically evaluate the safety and potential adverse effects of PEMF therapy.

## 5. Conclusions

In conclusion, this systematic review finds that the effect of PEMF therapy on KOA is nuanced and highly dependent on timing and device parameters. While we did not find a significant overall benefit for pain or function, our analysis revealed specific time-dependent effects, including early improvements in pain and later improvements in stiffness and daily activity. We also found that high frequency gives fast pain relief; high amplitude builds function. Although some of these effects were statistically significant, their magnitude did not consistently meet established thresholds for clinical meaningfulness. The primary challenge in this field is the profound heterogeneity of treatment protocols, which prevents a definitive conclusion. Therefore, while PEMF remains a promising non-invasive option, its role in clinical practice cannot be solidified without further large-scale, standardized research to identify optimal parameters and confirm long-term benefits.

## Figures and Tables

**Table 1:** Characteristics of the included studies.

First Author, Year	Participants (E/C)	PEMF (Amplitude/Frequency)	Outcomes
Wuschech, 2015 [28]	44/13 (only 42 subjects were included in the analysis of WOMAC-total and WOMAC-activity)	105 mT/4–12 Hz	WOMAC
Bagnato, 2016 [22]	30/30	0.0001 mT/1000 Hz	WOMAC, VAS pain score (0–100)
Dündar, 2016 [29]	20/20	0.1 mT/50 Hz	VAS pain score (0–100)
Elboim-Gabyzon, 2023 [30]	20/20	10 mT/30 Hz	WOMAC, VAS pain score (0–10), TUG
Yabroudi, 2024 [31]	17/17	5 mT/50 Hz	FTSST
Wang, 2024 [23]	30/30	1 mT/50 Hz	VAS pain score (0–10), FTSST
Pasin, 2025 [32]	30/30	10 mT/30 Hz	WOMAC, VAS pain score (0–10), TUG
Comino-Suárez, 2025 [33]	20/20	10 mT/50 Hz	WOMAC, VAS pain score (0–10), TUG
Ehsan Hashemi, 2025 [34]	34/32	1.2 mT/10–100 Hz	WOMAC, VAS pain score (0–100)

E, Experimental; C, Control; PEMF, Pulsed Electromagnetic Field; WOMAC, Western Ontario and McMaster Universities Osteoarthritis Index; VAS, Visual Analog Scale; TUG, Timed Up and Go; FTSST, Five Times Sit-to-Stand Test.

## Data Availability

Data are provided within the manuscript or Supplementary Materials files.

## Supplementary Materials

The following supporting information can be downloaded at: https://www.mdpi.com/article/10.3390/medicina62040677/s1, Table S1: PRISMA Checklist; Table S2: Keywords and search results in different databases; Table S3: Excluded studies and reasons.

## Abbreviations

The following abbreviations are used in this manuscript:

KOA	Knee osteoarthritis
PEMF	Pulsed electromagnetic field
RCTs	Randomized controlled trials
VAS	Visual Analog Scale
WOMAC	Western Ontario and McMaster Universities Osteoarthritis Index
OA	Osteoarthritis
GBD	Global Burden of Disease
OARSI	Osteoarthritis Research Society International
ACR	American College of Rheumatology
AF	Arthritis Foundation
EULAR	European Alliance of Associations for Rheumatology
TUG	Timed Up and Go
FTSST	Five Times Sit-to-Stand Test
ROB	Risk-of-Bias
RevMan	Review Manager
MD	Mean difference
CI	Confidence interval
SD	Standard deviation
MCID	Minimal clinically important difference
